# An Image-Based Class Retrieval System for Roman Republican Coins

**DOI:** 10.3390/e22080799

**Published:** 2020-07-22

**Authors:** Hafeez Anwar, Serwah Sabetghadam, Peter Bell

**Affiliations:** 1 Interdisciplinary Center for Digital Humanities and Social Sciences, Friedrich-Alexander University, 91052 Erlangen, Germany; 2 Department of Electrical and Computer Engineering, COMSATS University Islamabad Attock Campus, Attock City 43600, Pakistan; 3 Institute of Software Technology and Interactive Systems, Vienna University of Technology, 1040 Vienna, Austria; sabetghadam@ifs.tuwien.ac.at

**Keywords:** image entropy, image processing, image classification

## Abstract

We propose an image-based class retrieval system for ancient Roman Republican coins that can be instrumental in various archaeological applications such as museums, Numismatics study, and even online auctions websites. For such applications, the aim is not only classification of a given coin, but also the retrieval of its information from standard reference book. Such classification and information retrieval is performed by our proposed system via a user friendly graphical user interface (GUI). The query coin image gets matched with exemplar images of each coin class stored in the database. The retrieved coin classes are then displayed in the GUI along with their descriptions from a reference book. However, it is highly impractical to match a query image with each of the class exemplar images as there are 10 exemplar images for each of the 60 coin classes. Similarly, displaying all the retrieved coin classes and their respective information in the GUI will cause user inconvenience. Consequently, to avoid such brute-force matching, we incrementally vary the number of matches per class to find the least matches attaining the maximum classification accuracy. In a similar manner, we also extend the search space for coin class to find the minimal number of retrieved classes that achieve maximum classification accuracy. On the current dataset, our system successfully attains a classification accuracy of 99% for five matches per class such that the top ten retrieved classes are considered. As a result, the computational complexity is reduced by matching the query image with only half of the exemplar images per class. In addition, displaying the top 10 retrieved classes is far more convenient than displaying all 60 classes.

## 1. Introduction

**Figure 1:**
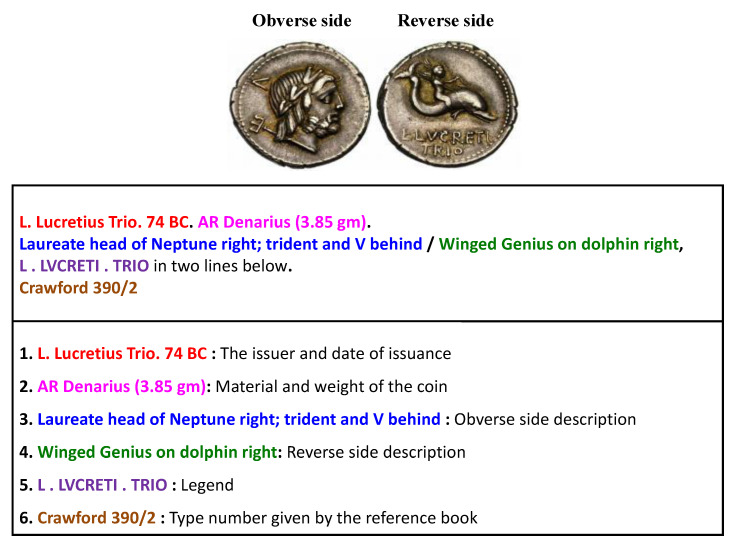
Description of an ancient coin.

We propose a holistic image-based class retrieval system for ancient Roman Republican coins that can be instrumental for a number of non-expert audience such as museum visitors, Numismatics students, hobbyists, auctions Websites, as well as the art market in general. For instance, most of the museums visitors are completely unaware about the historic context of the displayed coins. Such scholarly information about the ancient coins is only available in standard reference books [1], where the coins are indexed according to the “*coin type number*” that we call “*class*” in the rest of paper. For a given coin class, this information includes the issuer, date of issuance, information about the material of coin, and the complete description of obverse and reverse motifs. An example of such is shown in Figure 1 for a Roman Republican coin of type number “Crawford 390/2”. Our proposed system aims at the retrieval of such information when provided with an image of the reverse side of a Roman Republican coin. However, for such retrieval, as a first step, the provided coin image is assigned a type number or class which is done by a specialized image-based classification framework that uses methods from the field of computer vision.

**Figure 2:**
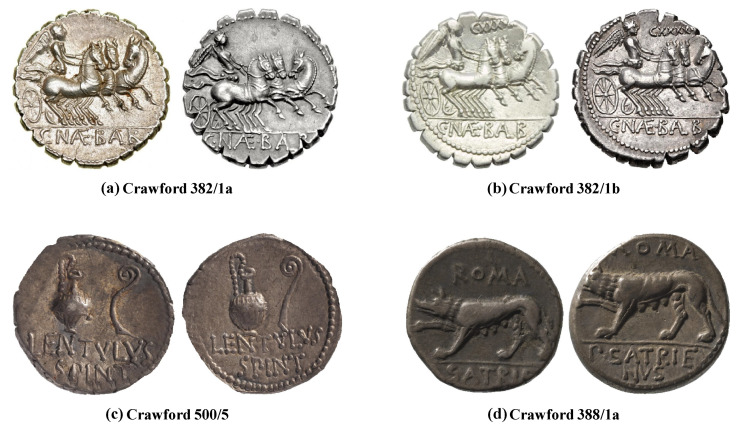
Variations in examples of the same coin type.

Nonetheless, the classification of ancient coins based on their images is a challenging task due to variations caused by several factors. A main problem is the high intra-class and low inter-class variations due to large number of coin classes. In case of Roman Republican coins, there are 550 classes with more than 1000 subclasses [1]. The reverse motifs of coins in Figure 2a,b show the same object: the triga (a chariot with three horses). However, the two classes can only be distinguished from each other based on the legend above triga resulting in minimal inter-class variations. Similarly, the dies used to manufacture coins of the same class can differ resulting in dissimilarities among these coins. For instance, the structures of jug and lituus differ in both examples shown in Figure 2c. The inconsistency in visual appearance of ancient coins is caused by their age and preserving conditions. Due to these reasons the visually discriminating parts of ancient coins are worn out creating dissimilarities among coins of the same class. For instance, Figure 2d shows reverse motifs of the same coin class where the main object is a wolf. However, on one of the motifs, head of the animal is missing. Finally, the imaging conditions also cause variations in coin images such as those caused by nonuniform illuminations.

## 2. Status of Research

Due to the aforementioned challenges, the image-based methods proposed for the classification of modern day coins are not adaptable for ancient coins [2]. The methods that deal with the image-based classification of ancient coins are either use machine learning algorithms [3] or local featurse matching [4,5]. However, some of the methods propose to use image-based recognition of certain parts of ancient coins for their classification. For instance, Anwar et al. [6,7] propose to perform image-based classification of ancient Roman Republican coins by recognizing their reverse motifs. Various objects, people, or scenes of historic events are minted as main motifs on the reverse sides. However, some of these motifs may depict the same objects with modifications in their styles and accompanying legends and by-marks. This makes the reverse motifs-based classification coarse-grained that can further be refined with the help of other recognition schemes such as legends recognition [8,9]. A legend carries rich information about a given coin type due to which it becomes the most suitable and natural choice for coin classification. However, legend is also the most vulnerable visual part of a coin that gets degraded due to wear and tear. This makes it less efficient and helpful to achieve a better performance in image-based classification frameworks [10]. The image-based obverse side portraits recognition [3,11] is also proposed for ancient coins classification. However, these methods are only evaluated on the images of Roman Imperial coins. A large portion of Roman Republican coins depict the portrait of Roma due to which, the obverse side portrait recognition is non-practical for image-based class recognition of Republican coins. Finally, the recent state-of-the-art deep learning frameworks are also utilized for ancient coin classification [11,12,13].

Nonetheless, all these methods use one or the other form of image classification where the task is to classify a given coin image into one of the coin classes. We extend this concept a step further by showing the class relevant information from the expert sources in a user friendly manner. This is due to the fact that the knowledge about an ancient coin is also of utmost importance when it comes to particular communities such as archaeologist, students and researchers. Consequently, the proposed system has a potential to be used as an assisting or educational tool at a number of places such as museums, educational institutes, or even online auction websites.

## 3. Roman Republican Coin Image Dataset

The database consists of 60 coin classes where each class is represented by 10 exemplar images of their reverse side motifs. The dimensions of all the images are 150×150 and they have the following properties.

- The exemplar coin images of any given class differ from one another mainly due to the non-rigid deformations caused by wear and tear on the coin.
- All the coins are imaged with a homogeneous background.
- The coin is depicted at the image center in almost all of the images.
- As the coins belong to the Roman Republican era, the scale variations in the coin images remains negligible.
- All the coins are imaged with their canonical orientations resulting in negligible variations caused by rotations.

## 4. Proposed System

**Figure 3:**
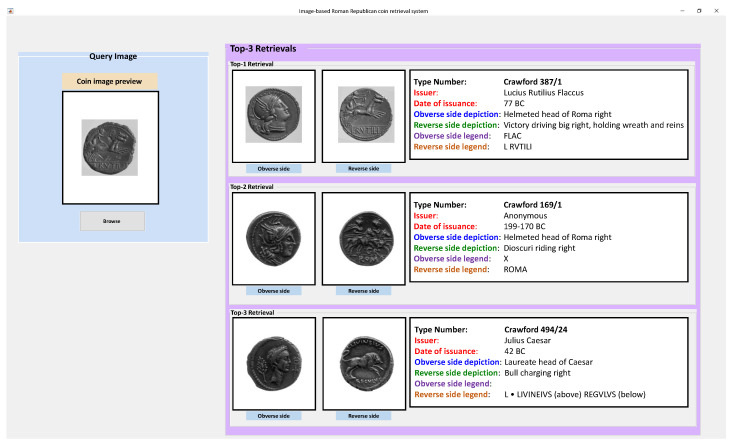
Graphical user interface (GUI) of the proposed system.

The graphical user interface (GUI) of our proposed holistic system for image-based classification of Roman Republican coins is shown in Figure 3. It provides a convenient interface where a single coin image of reverse side is provided to the system. After performing online matching and ranking, the most relevant coin classes are shown in the GUI along with their information from the standard source.

**Figure 4:**
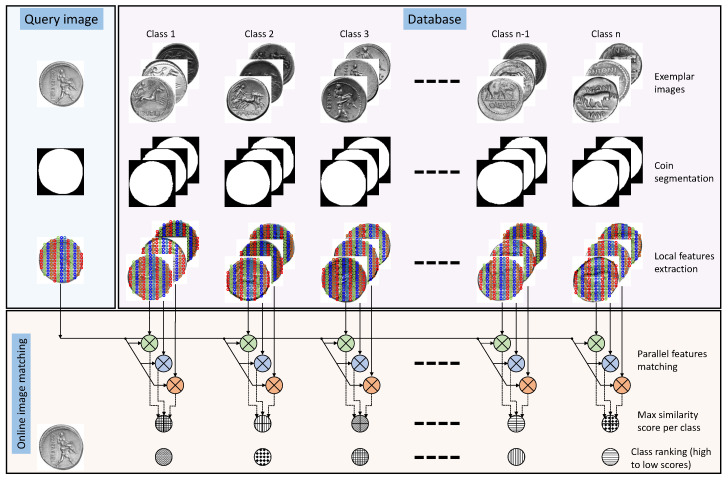
Methodology of the proposed system.

Figure 4 shows the exemplar images for each class in the database where only three images per class are depicted. In this figure we show all the steps of our methodology as follows:

### 4.1. Step 1: Coin Image Segmentation

We use automatic coin segmentation [14] to extract the image region that depicts the coin. This method is specifically designed for ancient coins where as a first step, it employs the local entropy and range filters. The responses of both these filters are then combined and the image binarization is done using a confidence score that is maximum for circular objects. As the shape of a coin is near circular, it is successfully segmented from the background. In this way, the method also ensures scale- and translation-invariance. The segmentation masks for exemplar images generated using this method are shown in Figure 4.

### 4.2. Step 2: Local Features Extraction

The local image patches are densely sampled and then represented with local feature descriptors. In the proposed framework, we use the Local Image Descriptor Robust to Illumination Changes (LIDRIC) [15], which is insensitive to changes in illumination for textureless object such as the ancient coins. The local features for the exemplar coin images are extracted and stored in the database as shown in Figure 4.

### 4.3. Step 3: Parallel Features Matching

For a query image, the segmentation and features extraction are performed as stated previously. The next step is to match the local features of the query image with those of the exemplar images in the database. A depicted in Figure 4, we match local features in a parallel manner by using a matching strategy that takes information about their geometric plausibility [5].

### 4.4. Step 4: Maximum Similarity Score Calculation for Each Coin Class

The matching assigns similarity scores to each exemplar image of all the coin classes where a higher score shows a high similarity. In this way, for each coin class, similarity scores are determined from its exemplar images. However, a “*max*” operation is then performed on these 10 values, where the maximum score is considered for that particular coin class. Consequently, for a query image, the number of similarity scores reduces to 60; one per coin class.

### 4.5. Step 5: Class Ranking Based on Similarity Scores

Finally, all the 60 classes are ranked in a descending order based on their similarity values where the one with highest similarity is assigned to the query image. However, our system aims at the depiction of candidate classes for the query image along with their information from the standard Numismatics source leading to the depiction of top “N” classes in the GUI.

## 5. Experiment Design and Results

For a query image, the relevant classes are found by matching it with exemplar images of each class. As it is an online image matching process, the cost of computations must be optimized by evaluating the number of matches per class. For instance, in the worst case, each class has 10 exemplar images and there are a total of 60 classes resulting in 600 image matchings. This will ultimately increase waiting time in our proposed framework due to which, we evaluate the number of matches per class. Furthermore, for the query image, all the 60 classes will be ranked from top to bottom with respect to their similarity scores and consequently will be displayed in the GUI. However, displaying all the classes along with their information will cause inconvenience for the users. Therefore, another parameter that is worth investigating is the number of retrieved classes to be displayed in the GUI.

For the number of matches, we start by comparing the query image with two randomly selected images of each class. We repeat this experiment by increasing the number of randomly selected images to three, four and up to nine. Through this procedure, we find the optimal number of matches both in terms of classification accuracy and time. For instance, in case of experiment for comparing with two random images from each class, we have a total of 2×60 images as our exemplar image pool. This results in matching the query image to a set of 120 exemplar images. In this way, we get the candidate coin classes for a query image ranked in the order of their relevance. However, we only consider the top ten relevant classes and display their representative images and descriptions in the GUI from top to bottom.

**Figure 5:**
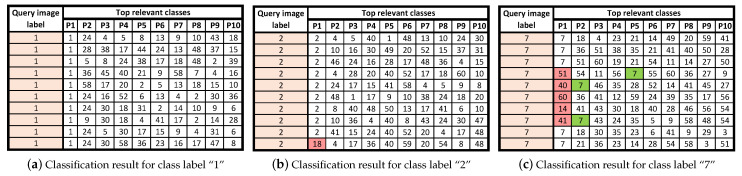
Top-10 most relevant classes retrieved for a query images belonging to classes “1”, “2”, and “7”.

A few examples of such ranking with query images from classes “1”, “2”, and “7” are shown in Figure 5a–c, respectively. In each row, the first column shows the label of a query image while the other columns show most relevant retrieved class labels for this query image. It can be observed that for all the ten query images of class “1”, the top relevant retrieved labels are exactly the same while in case of class “2” only the label for the last query image is not a correct one. However, in case of class “7”, the retrieved class labels for multiple query images are either not within the top ten or they are ranked at various positions within the top ten other than the first position. Nonetheless, it shows that the retrieved label accuracy is more likely to increase if we extend the search space for top retrieved labels, for instance in this case, the top ten most relevant labels are considered.

**Figure 6:**
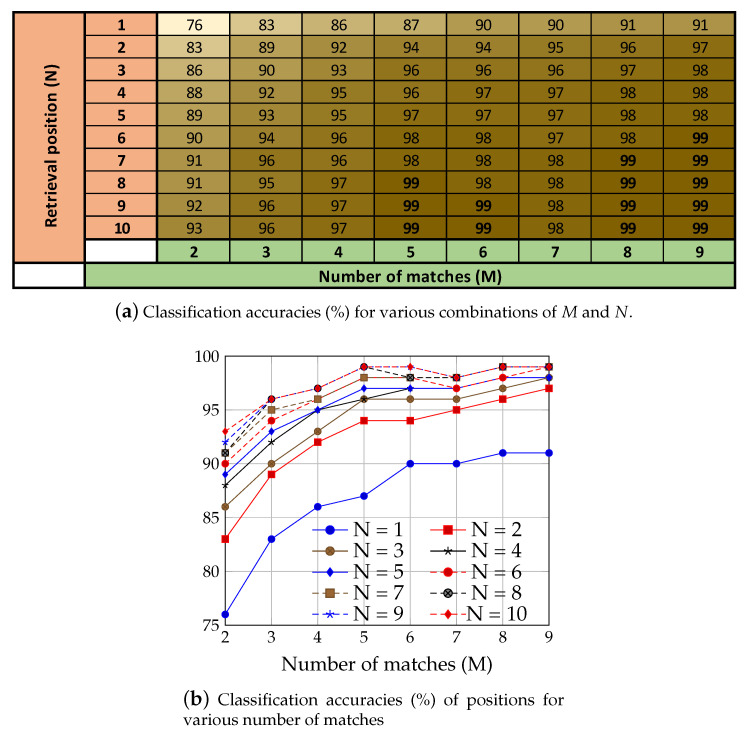
Classification accuracies (%) at various values of *M* and *N*.

We carried out elaborated experiments on the relationship of the number of matches denoted by *M* and the top most relevant retrieved classes denoted by *N*. The number of matches are empirically selected as M∈{2,3,…,9} and N∈{1,2,…,10}. For instance, the value of M=2 means that a given query image is matched with randomly selected two exemplar images from each class. As the selection of exemplar images is done randomly for matching, for each value of *M*, the experiments are performed 5 times and an average classification accuracy is reported. Similarly, for a given query image, all the 60 coin classes are ranked based on their achieved similarity scores from high to low. The correctly predicted label for a query image can be at any position among those 60 labels. However, for displaying, we search for least number of retrieval positions *N* where highest possible classification accuracy is also achieved. For instance, the value N=1 means that only the first predicted label with highest similarity score is considered. Similarly, the value of N=2 means that the relevant label of a given query image can be either at the first position or at the second. In this way, we go up to 10 positions of the retrieved class labels and report their accumulative classification accuracy. The average classification rates achieved by various combination of *M* and *N* are shown in Figure 6a. Figure 6b shows a summarized view of the values from which the following conclusions can be drawn.

1. An overall increase in classification accuracy is observed by increasing both *M* and *N*.
2. The lowest accuracy is achieved by considering N=1 for all number of matches.
3. The highest classification accuracy of 99% is achieved for M>=5 and N>=7. However, an exception exists at M=7 that achieves the highest classification accuracy of 98%.
4. In order to reduce the computational time, the value of *M* should be 5 and that of *N* should be 10.

To summarize, our proposed framework retrieves most relevant classes for a given query image by matching it with five randomly selected class exemplar images stored in the database. This results in a total of 300 (5×60) matches. The matched images will then be ranked in descending order based on their achieved similarity scores. Finally, they are replaced by their representative coin classes where the top 10 unique coin classes will be displayed in the GUI.

We also performed experiments to show the time taken by matching a query image with various number of class exemplar images. The matching is performed using the parallel architecture of an Intel^®^ Core™ i7-8700K CPU of 3.70GHz with 12 threads in Matlab^®^ R2018b. The experiments are repeated 20 times and the average time taken by each setting along with the standard deviation is shown in Table 1.

**Figure 7:**
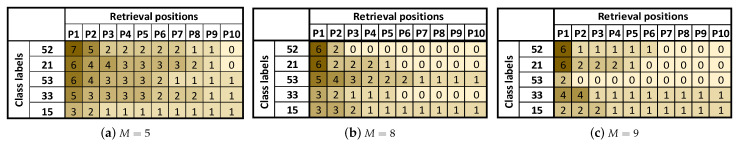
Ranking of classes based on the number of misclassified query images for 5, 8, and 9 matches and at various retrieval positions.

Figure 7 shows the top five challenging coin classes along with the number of their misclassified query images for M=5,8,and9 and for the top-10 retrieval positions. It can clearly be observed that the number of misclassifications decrease with the increase in search space for coin class up to 10 positions.

## 6. Conclusions

We proposed an image-based framework for class retrieval of the Roman Republican coins. It takes a query image as an input, classifies using exemplar-based image classification, and then displays the resulting candidate top 10 classes in the GUI along with their information from the standard reference book. Our proposed framework can become an interesting application tool for a wide range of users such as museum visitors, Numismatics students and researchers, hobbyists, and online auction websites. We thoroughly evaluated various parameters of the framework such as number of exemplar images per class to which the query image will be matched and the number of top relevant classes to be displayed in the GUI. From our experiments, we found that the five images per class and a search space for the relevant retrieved classes extended up to 10 position successfully classifies the query image with 99% accuracy.

In future, we plan to extend our database to span more coin classes, shift the current matching algorithm to GPU-based computations in order to meet the real-time matching, and include more state-of-the-art methods for image matching. 

## Figures and Tables

**Table 1:** Time taken in seconds by comparing a query image with various number of exemplar images; mean and std for 20 runs.

	Number of Image Matches Per Class							
	2	3	4	5	6	7	8	9
Mean	4.26	6.03	7.97	10.24	12.36	14.55	16.56	18.26
Std	0.24	0.55	0.66	0.98	1.14	1.44	1.47	1.45
